# Prospective observational study to evaluate the feasibility of the mobile app for mild cognitive impairment detection and screening

**DOI:** 10.3389/fdgth.2025.1535900

**Published:** 2025-02-07

**Authors:** Reo Hamaguchi, Seiji Hongo, Naoto Doi, Hisamitsu Ide, Ryozo Saito, Junji Kishimoto, Nobuhiro Handa, Shigeo Horie

**Affiliations:** ^1^ Department of Digital Therapeutics, Graduate School of Medicine, Juntendo University, Tokyo, Japan; ^2^ Nanko Clinic of Psychiatry, Fukushima, Japan; ^3^ Ichigaya Himorogi Clinic, Tokyo, Japan; ^4^ Life Quest Inc., Tokyo, Japan; ^5^ Center for Clinical and Translational Research, Kyushu University Hospital, Fukuoka, Japan

**Keywords:** mobile app, smartphone app, neuropsychological tests, cognitive disorders, mild cognitive impairment, Alzheimer's disease, dementia

## Abstract

**Introduction:**

The increasing prevalence of dementia in aging populations necessitates effective and accessible cognitive screening tools. This study aimed to evaluate the feasibility and reliability of a newly developed mobile app for detecting and screening mild cognitive impairment (MCI).

**Methods:**

The mobile app, developed by LifeQuest Co., Ltd. (Minato-ku, Tokyo), is an original tool inspired by the Japanese version of the Montreal Cognitive Assessment (MoCA-J). A prospective observational study was conducted with 20 participants, including healthy individuals, MCI patients, and those with mild to moderate-severe dementia. Participants completed both the mobile app and the MoCA-J in a randomized order within a two-week period, with a minimum one-day interval between tests.

**Results and conclusion:**

The intraclass correlation coefficient (ICC) between the mobile app and the MoCA-J was 0.956 (95% CI: 0.89-0.983), demonstrating a very high level of correlation. All participants successfully completed the mobile app assessment, highlighting its feasibility across various cognitive levels. Although minor technical issues and usability challenges were identified, the results support the mobile app as a reliable and user-friendly alternative for cognitive screening. Further studies with larger sample sizes are necessary to validate these findings and refine the app for broader clinical use.

## Introduction

The prevalence of dementia in Japan is increasing with the advancement of an aging society. According to data from the Hisayama Study, the estimated prevalence of dementia among individuals aged 65 years and older is projected to reach approximately 20% by 2025, with an estimated 7 million individuals affected (1). While dementia treatment often combines pharmacological and non-pharmacological therapies to alleviate symptoms of cognitive decline and behavioral and psychological symptoms of dementia (BPSD), there is no established treatment to improve or halt the progression of dementia itself. However, recent developments in pharmacological treatments have shown promise. Lecanemab and donanemab, both anti–β-amyloid monoclonal antibodies, have been developed to reduce cognitive impairment and amyloid burden in patients with early Alzheimer's disease (AD) (2–4). The prevalence of mild cognitive impairment (MCI), a precursor stage to dementia, is reported to be 15%–25% among the elderly aged 65 and older (5–7). Studies indicate that 5%–30% of individuals with MCI progress to dementia annually (8, 9), while 15%–40% of MCI cases may revert to normal cognitive function each year (10–12). Therefore, early detection and intervention at the MCI stage are considered crucial for dementia prevention.

Various neuropsychological tests are conducted for the diagnosis and assessment of dementia and MCI. The Mini-Mental State Examination (MMSE) is widely used internationally in clinical practice and research for cognitive screening. In Japan, the Hasegawa's Dementia Scale-Revised (HDS-R) is also commonly utilized in clinical settings. The Montreal Cognitive Assessment-Japanese version (MoCA-J) is particularly useful for detecting mild dementia and MCI. Additionally, the Clinical Dementia Rating (CDR) scale is used as an indicator of dementia severity, and the Alzheimer's Disease Assessment Scale cognitive subscale Japanese version (ADAS-Jcog) is employed to evaluate the progression of AD symptoms. While these are established methods for assessing cognitive function, they require face-to-face evaluation by physicians or clinical psychologists, which presents a challenge in terms of convenience.

Recently, the field of digital health, utilizing smartphones and digital technology, has gained attention and is being increasingly applied to elderly individuals, including those with MCI (13). For instance, the ADAS-Jcog requires a long duration (over 45 min) and skilled examiners, but the Touch Panel-type Dementia Assessment Scale (TDAS) has been developed to allow measurement using a touchscreen on a computer, demonstrating potential as a substitute for ADAS-Jcog (14). The CogState Brief Battery, a computerized multitask test assessing attention, processing speed, memory, and executive function, has shown utility in detecting MCI (15). Similarly, Cognivue is a computerized tool for automatic cognitive assessment, conducting a 10-minute automated test measuring visuomotor, perceptual processing, and memory processing, reported to be useful as an adjunct tool for evaluating cognitive impairment (16). Moreover, simple cognitive assessment tools utilizing eye-tracking technology and short task movies and images have been developed (17). These tasks are designed to assess neurological domains such as deductive reasoning, working memory, attention, and recall, and have shown correlations with other neuropsychological test results (18).

Thus, various computerized cognitive tests have been developed as potential substitutes for traditional neuropsychological tests, showing promising utility. In this study, we evaluate a mobile app for MCI detection and screening developed by LifeQuest Co., Ltd. (Minato-ku, Tokyo). The app is designed for assessing cognitive function, detecting and screening for MCI and AD, and includes original tests inspired by the MoCA-J but developed as a standalone tool, allowing self-administration on a mobile device. The aim of this study is to investigate the correlation between the app's results and those from the MoCA-J.

## Participants and methods

### The mobile app for MCI detection and screening

**Figure 1:**
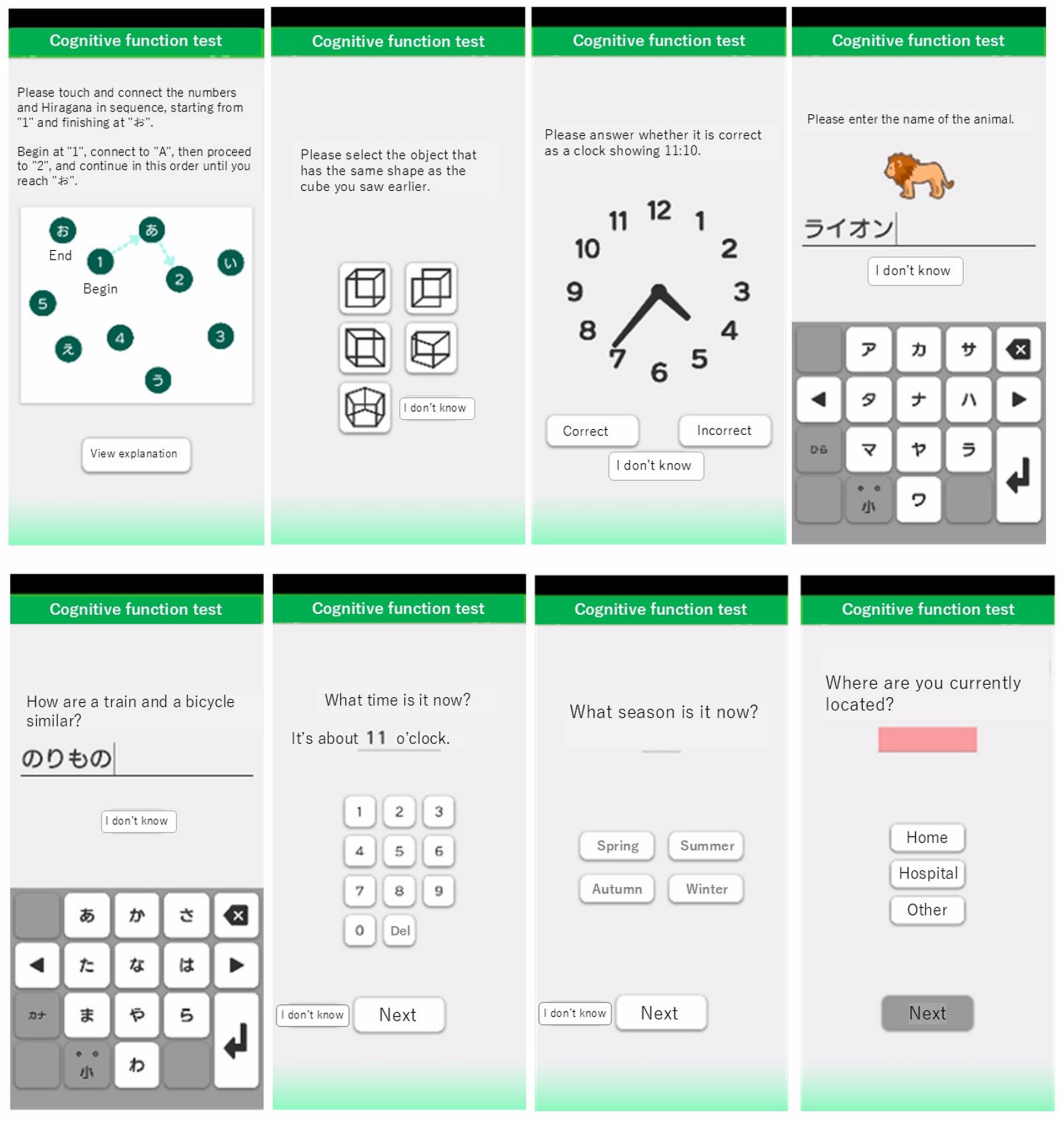
Screenshots of the mobile app for MCI detection and screening. Screenshots illustrating the interface of the app used for MCI detection and screening. The app includes various cognitive tasks inspired by the MoCA-J, with modified response formats and some question variations.

The original MoCA is a widely used cognitive screening tool designed to detect MCI and early AD (19, 20). The MoCA-J has been adapted to account for cultural and linguistic differences, ensuring its validity and reliability in Japanese populations (21). The MoCA-J consists of various cognitive tasks that assess different domains such as memory, visuospatial abilities, executive functions, attention, concentration, language, and orientation to time and place. It is traditionally administered in a face-to-face setting by a trained examiner. The mobile app for MCI detection and screening used in this study was developed by LifeQuest Co., Ltd. (Minato-ku, Tokyo) and images of this app are shown in Figure 1. This app, which includes original tests inspired by the MoCA-J, is designed for the assessment of cognitive function, as well as the detection and screening of MCI and AD. Responses entered into the app can be viewed on a web-based results screen, and the scores are automatically calculated. The questions in the app are electronically answered and were developed inspired by the principles of the MoCA-J. This app was not developed as a digital version of the MoCA-J but rather as an original tool inspired by its principles, with minor modifications tailored for mobile use. While the response format differs and some questions have added or have variations, a detailed comparison with the MoCA-J is provided in Table 1.

**Table 1:** Comparison of test items between MoCA-J and Mobile App for MCI detection and screening.

No.	Test item	MoCA-J	Points	Mobile App for MCI detection and screening	Points
1	Trail making	Drawing lines	1	Tap input	1
2	Visuospatial function (Cube)	Drawing a cube	1	– Multiple choice – ^a^Draw a cube on screen	1
3	Visuospatial function (clock)	Drawing a clock	3	Multiple choice	3
4	Naming (animals)	Oral response	3	Text input	3
5	Memory (5 words)	Oral response	None	Text input	None
6	Attention		(6)		(6)
	– Forward/backward digit span	– Oral response	2	– Numeric input	2
	– Vigilance	– Clapping hands	1	– Screen tapping	1
	– Serial subtraction	– Oral response	3	– Numeric input	3
7	Repetition	Oral response	2	Oral response	2
8	Verbal fluency (Words starting with “ka”)	Oral response	1	Text input	1
9	Abstraction	Oral response	2	Text input	2
10	Delayed recall (5 Words)	Oral response	5	Text input	5
11	Orientation		(6)		(6)
	– Year/month/date	– Oral response	1/1/1	– Numeric input	0.75/0.75/0.75
	– Day of the week	– Oral response	1	– Select day	0.75
	– City	– Oral response	1	– Text input	0.75
	– Place	– Oral response	1	– Select from home/hospital/others (GPS auto-detection)	0.75
				^b^Additional questions:	
				– Time: Numeric input	0.75
				– Season: Select season	0.75
Total^c^			30		30

^a^ Drawing a cube on the screen with a finger (performed for reference and not reflected in the score).

^b^ Additional questions: time and season. Since there are a total of 8 questions, each question is calculated as 0.75 points.

^c^ If the years of education are 12 or fewer, add 1 point (maximum score is 30).

#### Study design

**Figure 2:**
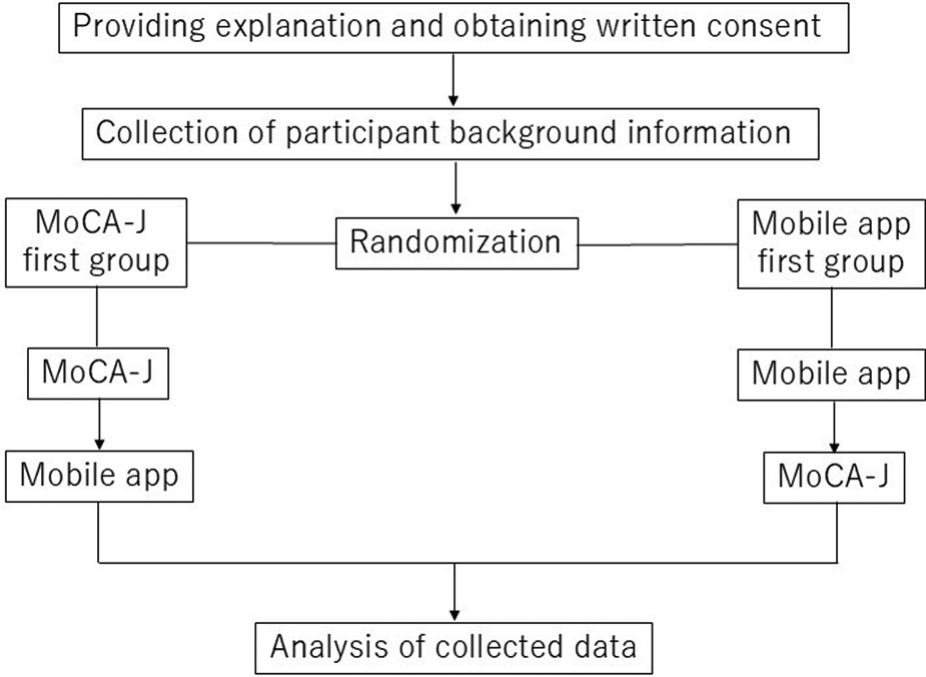
Flowchart of this study. Flowchart outlining the study design, including participant recruitment, randomization, and the administration of cognitive assessments.

This study was conducted as a prospective observational study aimed to evaluate the correlation between the mobile app for MCI detection and screening, and the MoCA-J. This study has been registered at the UMIN Clinical Trials Registry as UMIN000052091 on September 4, 2023. Following the acquisition of informed consent and collection of participant background information, the cognitive assessments (both the mobile app and the MoCA-J) were randomly administered in either of the following sequences: MoCA-J followed by the mobile app, or mobile app followed by the MoCA-J. The mobile app assessments were conducted in a clinical setting under supervision. These assessments were conducted within a two-week period, with at least one day in between each test (Figure 2).

#### Participants

Participants were enrolled from three institutes between September 28, 2023, and March 31, 2024. Participants included eight healthy individuals without a history of cognitive decline from LifeQuest Co., Ltd. Additionally, four participants each with MCI, mild dementia, and moderate to severe dementia were recruited from Ichigaya Himorogi Clinic, and Nanko Clinic of Psychiatry, respectively, totaling 12 participants. The total number of participants was set at 20. The classifications were based on the MMSE scores as follows; MCI: MMSE score 24–27, Mild dementia: MMSE score 21–23, Moderate to severe dementia: MMSE score ≤20.

#### Eligibility criteria

Inclusion Criteria:

 (1) Individuals aged 20–90 years at the time of enrollment
 (2) Participants or their families who have provided informed consent after receiving sufficient explanation about the study

Exclusion Criteria:

 (1) Participants who have undergone the MoCA-J within one month prior to the scheduled study date
 (2) Individuals with visual or auditory impairments, ataxia, or other disabilities that prevent the use of a smartphone
 (3) Participants deemed unsuitable for the study by the investigators

#### Study endpoints

Primary Endpoint:

 (1) The proportion of participants who successfully complete the mobile app for MCI detection and screening

Secondary Endpoints:

 (1) The correlation between the total scores and corresponding item results of the mobile app and the MoCA-J
 (2) The correlation between the respective scores of the mobile app and the MoCA-J, comparing the group that completed the mobile app first with the group that completed the MoCA-J first

#### Statistical analysis

Descriptive statistics were conducted on participant demographics collected via questionnaire, including age, gender, body mass index (BMI), highest educational attainment, alcohol consumption, smoking habits, and current medical conditions. The correlation between the scores of the mobile app for MCI detection and screening, and the MoCA-J was evaluated using intraclass correlation coefficients (ICC), and scatter plots were generated. Additionally, the agreement between the individual item results of the mobile app and MoCA-J was assessed using the kappa coefficient. The correlation between the respective scores of the mobile app and the MoCA-J, comparing the group that completed the mobile app first with the group that completed the MoCA-J first, was analyzed using the unpaired t-test.

## Results

### Patient characteristics

A total of 20 participants (11 male and 9 female), who were categorized into four groups: healthy individuals (*n* = 8), patients with MCI (*n* = 4), patients with mild dementia (*n* = 4), and patients with moderate to severe dementia (*n* = 4), were enrolled in the study period. The demographic and clinical characteristics of the study participants are summarized in Table 2. Healthy participants had a mean age of 49.8 years, while the other groups had mean ages of 73.5 years (MCI), 80.6 years (mild dementia), and 78 years (moderate to severe dementia). It should be noted that the healthy group was younger than the other groups, which may affect the comparison of mobile app usability. All healthy participants had more than 12 years of education, while most participants in the other groups had 12 years or less. Among the comorbidities, hypertension was common, being present in 37.5% of the healthy group, 25% of the MCI group, 50% of the mild dementia group, and 75% of the moderate to severe dementia group.

**Table 2:** Patients’ characteristics.

	No. of patients (%)				
	Healthy (*n* = 8)	MCI (*n* = 4)	Mild dementia (*n* = 4)	Moderate to severe dementia (*n* = 4)	All (*n* = 20)
Age, years					
Mean (range)	49.8 (38–72)	73.5 (66–82)	80.6 (73–85)	78 (73–87)	66.4 (38–87)
Gender					
Male	4 (50)	3 (75)	3 (75)	1 (25)	11 (55)
Female	4 (50)	1 (25)	1 (25)	3 (75)	9 (45)
BMI					
Mean (range)	22.5 (17.5–29.4)	22.6 (19.8–26.7)	20.1 (15.2–24.1)	24.7 (19.1–30.3)	22.5 (15.2–30.3)
Education					
12 years <	8 (100)	1 (25)	2 (50)	1 (25)	12 (60)
≤12 years	0 (0)	3 (75)	2 (50)	3 (75)	8 (40)
MMSE score					
Mean (range)	NA	24.3 (24–25)	22.3 (21–23)	13.0 (4–16)	NA
Alcohol consumption					
Daily	3 (37.5)	1 (25)	1 (25)	0 (0)	5 (25)
2–3 times/week	2 (25)	0 (0)	0 (0)	0 (0)	2 (10)
Occasional	3 (37.5)	3 (75)	3 (75)	4 (100)	13 (65)
None	0 (0)	0 (0)	0 (0)	0 (0)	0 (0)
Smoking					
Current	1 (12.5)	1 (25)	0 (0)	0 (0)	2 (10)
Past	3 (37.5)	2 (50)	3 (75)	1 (25)	9 (45)
Never	4 (50)	1 (25)	1 (25)	3 (75)	9 (45)
Comorbidities					
Hypertension	3 (37.5)	1 (25)	2 (50)	3 (75)	9 (45)
Hyperlipidemia	0 (0)	2 (50)	0 (0)	0 (0)	2 (10)
Diabetes	0 (0)	1 (25)	0 (0)	0 (0)	1 (5)
Heart disease	0 (0)	0 (0)	0 (0)	0 (0)	0 (0)
Stroke	0 (0)	0 (0)	0 (0)	0 (0)	0 (0)
Depression	0 (0)	1 (25)	2 (50)	0 (0)	3 (15)
Parkinson's disease	0 (0)	0 (0)	0 (0)	0 (0)	0 (0)
Malignancy	0 (0)	0 (0)	0 (0)	0 (0)	0 (0)
Others	0 (0)	2^a^ (50)	3^b^ (75)	1^c^ (25)	6 (30)

^a^ Benign prostatic hyperplasia (BPH).

^b^ BPH, hyperuricemia.

^c^ Epilepsy.

### Completion of the mobile app for MCI detection and screening

All 20 participants successfully completed the mobile app for MCI detection and screening, regardless of their cognitive impairment level. However, one instance of a Wi-Fi connectivity issue caused a malfunction in the repetition task. The repetition task requires participants to orally repeat a sentence after hearing it, and the accuracy is determined using speech recognition on the mobile app. Due to the Wi-Fi issue, this process failed, and no score was recorded for this task.

### Correlation between mobile app and MoCA-J scores

**Figure 3:**
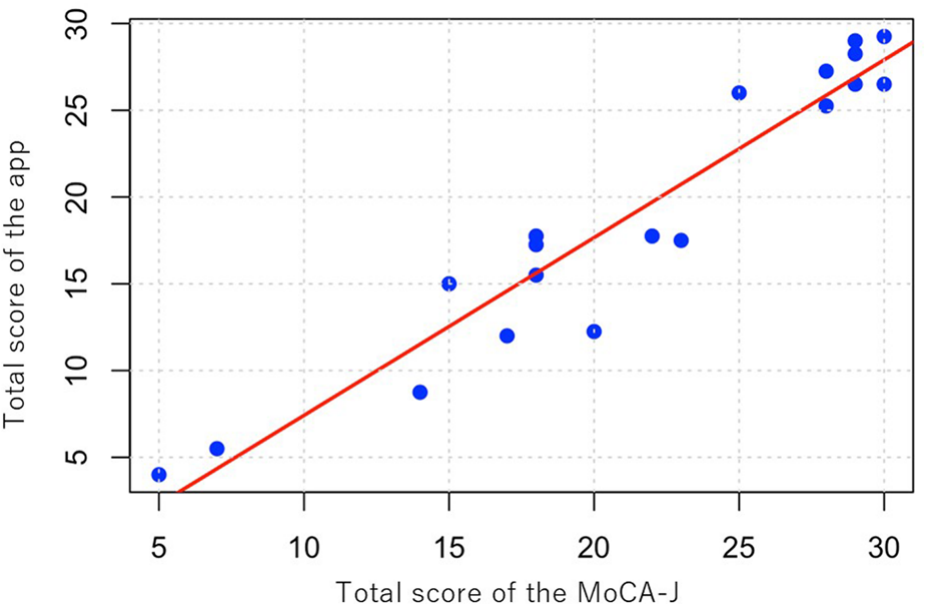
Scatter plot of total scores. Scatter plot illustrating the correlation between total scores obtained from the mobile app and the MoCA-J, with a trend line indicating their relationship (ICC = 0.956).

Table 3 presents the scores for each task and the total scores of both the mobile app and the MoCA-J, along with the kappa coefficients for each individual task. The ICC was 0.956 (95% CI: 0.89–0.983), indicating a very high degree of correlation between the mobile app for MCI detection and screening, and the MoCA-J. Additionally, Figure 3 provides a scatter plot illustrating the correlation between the two methods.

**Table 3:** Score of MoCA-J and mobile app for MCI detection and screening.

	MoCA-J (*n* = 20)	Mobile app for MCI detection and screening (*n* = 20)	Kappa/ICC^a^	*p*-value
Trail Making	0.75	0.75	0.467	0.0369
Visuospatial Function (Cube)	0.60	0.45	0.314	0.142
Visuospatial Function (Clock)	2.40	2.50	0.0741	0.646
Naming (Animals)	2.70	1.90	0.192	0.158
Memory (5 Words)	–	–	–	–
Attention				
Forward/Backward digit span	1.50	1.55	0.333	0.0576
Vigilance	0.95	0.80	0.348	0.0402
Serial subtraction	2.55	1.75	0.18	0.136
Repetition	1.00	1.11**	0.392	0.00694
Verbal Fluency (Words starting with “ka”)	0.50	0.35	0.70	0.00103
Abstraction	1.35	0.90	0.431	0.00114
Delayed Recall (5 Words)	2.40	2.40	0.692	5.94e–06
Orientation	4.65	4.61	0.0449	0.424
Total	21.75	19.01**	0.956	2.47e–11

^a^ Kappa coefficients are used for individual tasks, while the ICC is used for the total score.

** *n* = 19.

### Comparison of administration order on mobile app and MoCA-J scores

The scores of the mobile app and the MoCA-J were compared between the group that completed the mobile app first and the group that completed the MoCA-J first. An unpaired *t*-test showed no significant difference between the groups. For the mobile app, the first group had a mean score of 19.8 (SD 7.9), and the second group had a mean score of 18.3 (SD 8.8) (*p* = 0.700). For the MoCA-J, the first group had a mean score of 20.8 [standard deviation (SD) 8.0], while the second group had a mean score of 21.9 (SD 7.6) (*p* = 0.766). These results suggest that the order of test administration does not affect the assessment outcomes.

## Discussion

This study aimed to evaluate the feasibility and correlation of the mobile app for MCI detection and screening compared to the MoCA-J. All 20 participants successfully completed the mobile app, regardless of their cognitive impairment level. This indicates that the mobile app is generally user-friendly and accessible for individuals across a range of cognitive abilities. The correlation between the mobile app and MoCA-J was found to be very high (ICC = 0.956), suggesting that the mobile app is a reliable alternative to the MoCA-J. This high correlation indicates that the mobile app can effectively measure cognitive function in a manner comparable to the MoCA-J.

Recently, lecanemab and donanemab, both anti–β-amyloid monoclonal antibodies, were approved for use in individuals with MCI and early AD (2–4). However, identifying eligible candidates for anti–β-amyloid monoclonal antibody treatment remains a significant challenge due to the rigorous eligibility criteria. A population-based study revealed that only 8% of individuals with MCI or mild dementia meet the clinical trial criteria (22). Furthermore, it is uncommon for individuals to seek medical attention during the early stages of cognitive impairment, making the detection of MCI or early AD challenging in real-world clinical settings. In this context, our mobile app for MCI detection and screening, which demonstrates a high correlation with the MoCA-J, may offer a promising solution. This app has the potential to enable effective screening for potential MCI cases through self-assessment, reducing the burden on healthcare professionals.

Specific challenges with the mobile app were noted. A Wi-Fi connectivity issue affected the repetition task for one participant, highlighting a potential technical challenge that needs addressing. Specifically, the repetition task could not be scored due to the connectivity problem. In future updates, offline capabilities and enhanced error-handling features will be considered to ensure uninterrupted functionality and allow users to seamlessly retry tasks in the event of connectivity issues. There were difficulties with abstract questions because the predetermined correct answers were insufficient, leading to cases where correct responses were not recognized as such. Addressing this issue by expanding the range of acceptable answers is necessary. Furthermore, the verbal fluency task (words starting with “ka”) was found to be more challenging when entered via the mobile app compared to answering orally. This was particularly difficult for elderly participants who were less familiar with using smartphones. To improve usability, future developments will include refining the app's interface to make it more intuitive and user-friendly for older adults and expanding the range of acceptable responses for verbal and abstract fluency tasks. Improving these aspects in future updates will enhance the app's robustness and accessibility across diverse user groups.

Mobile applications and digital tools for cognitive assessments offer promising alternatives to traditional, clinic-based methods. These technologies enable frequent, brief, and repeated assessments in natural settings, enhancing patient-centered care and detection of subtle cognitive declines. Studies have shown the feasibility, reliability, and validity of mobile cognitive assessments, with adherence rates averaging 79.2% (23). Mobile technologies enable early detection of cognitive decline and support continuous monitoring and early intervention by providing convenient and cost-effective assessments (24). Eye-tracking (ET) technology is increasingly recognized as a valuable tool for assessing cognitive impairments (17). Research indicates ET's potential in diagnosing cognitive impairments and tracking decline over time, showing modest correlations with traditional cognitive assessments (25). Spontaneous speech analysis has gained attention for cognitive testing, using linguistic and acoustic features to detect cognitive impairments (26). Studies have shown that speech tempo, articulation rate, and pause patterns significantly differ between healthy individuals and those with cognitive impairments (27). The mobile app for MCI detection and screening used in this study is an original tool inspired by the MoCA-J. It incorporates tasks familiar to clinicians, which facilitates easier adoption and integration into existing clinical workflows. Compared to other methods, the app's design ensures user-friendliness and accessibility across cognitive levels. In addition, the app may contribute to the early detection of MCI, the reduction of healthcare costs through self-administered evaluations, and the provision of accessible cognitive screening for underserved populations. However, it is important to utilize a variety of assessment methods based on the specific situation to ensure comprehensive cognitive evaluation.

We acknowledge several limitations in this study. Firstly, the sample size was small, with only 20 participants, limiting the generalizability of the findings. Secondly, familiarity and proficiency with smartphone use varied among participants, particularly older adults, which may have influenced their performance on the mobile app for MCI detection and screening. Thirdly, the healthy group had a higher frequency of alcohol consumption compared to the other groups, which may have introduced an additional demographic imbalance. Lastly, while the mobile app was inspired by the MoCA-J and incorporated tasks based on its principles, it featured differences such as tap-based input instead of oral responses. These differences may have affected participants’ performance, particularly for those unfamiliar with such input methods, and highlight opportunities for refinement to improve user-friendliness and accessibility. In particular, the healthy group was younger than the other groups in this study. This age difference may have contributed to differences in familiarity with smartphone technology, potentially affecting the usability and performance results of the app across groups. Future studies should consider recruiting age-matched healthy controls and larger, more diverse cohorts to minimize these biases and enhance the generalizability of the findings.

## Conclusions

The mobile app for MCI detection and screening is a feasible and reliable alternative to the MoCA-J, with high correlation in scores. Despite some technical and usability challenges, it shows promise for clinical use. Further research and improvements are needed for full validation.

## Data Availability

The raw data supporting the conclusions of this article will be made available by the authors, without undue reservation.
